# Evaluation of Dietary Additives on Yellowtail Kingfish (*Seriola lalandi*) Gut and Skin Health and Disease Resistance Against *Photobacterium damselae* subsp. *damselae*

**DOI:** 10.1155/anu/8877734

**Published:** 2025-09-15

**Authors:** Md Reaz Chaklader, Lindsey Woolley, Chelsea Woods, Frances Stephens, Richard Smullen, Gavin Partridge

**Affiliations:** ^1^Department of Primary Industries and Regional Development, Fremantle 6160, Western Australia, Australia; ^2^Centre for Sustainable Aquatic Ecosystems, Harry Butler Institute, Murdoch University, Murdoch 6150, Western Australia, Australia; ^3^Independent Researcher, Middle Swan 6056, Western Australia, Australia; ^4^Ridley Agriproducts Pty Ltd., Robart Court, Narangba 4504, Queensland, Australia; ^5^Oceans Institute, University of Western Australia, Crawley 6009, Western Australia, Australia

**Keywords:** growth, immunostimulants, inflammatory cytokines, mucosal health, *Seriola lalandi*

## Abstract

The present study evaluated the effectiveness of five dietary additives in improving growth performance, gut and skin health and disease resistance against *Photobacterium damselae* subsp. *damselae* (Pdd) in yellowtail kingfish (YTK) (*Seriola lalandi*). The additives were top-coated onto a commercial diet. The five additives evaluated were brewer's yeast (BY), a yeast-based mannan oligosaccharide (MOS), a polyphenol product, a monoglyceride product and a combination of MOS and a superoxide dismutase (SOD) product. The monoglyceride and polyphenol products were trialled at both a low and high inclusion level. Diets were fed to YTK in triplicate tanks for 55 days. The results found that growth, feed utilisation and somatic indices were unaffected by additive inclusion. None of the additives affected the health of YTK liver, skin or the gut as determined by various histological metrics. These histological findings were supported by results of hindgut gene expression (*TNF-α*, *IL-10*, *Hsp70*, *mucin 2* and *i-mucin* and *SOD*), all of which were unchanged across the dietary treatments. Similarly, the additives did not stimulate the skin mucus quantity and skin mucosa barrier measurements (epidermis thickness and mucus cells), consistent with the results of relevant skin health gene expression measurements (*TNF-α*, *IL-8*, *calreticulin*, *Hsp70*, *mucin 2*, *CAT* and *SOD*). However, the MOS and low inclusion polyphenol treatments increased survival against Pdd challenge relative to control treatment. The immuno-modulating capacity of the polyphenol product was indicated by the absence of *Pdd* in faeces following a mucosal (bathe) challenge at the high inclusion; however, this did not translate to enhanced survival under systemic infection. However, further studies are needed to understand if optimisation of the inclusion levels of each additive might more strongly influence the health of YTK.

## 1. Introduction

Yellowtail kingfish (YTK) has been identified as Australia's next major aquaculture product due to its superior growth rate compared to Atlantic salmon (*Salmo salar*) and barramundi (*Lates calcarifer*), as well as its high market value [1]. The YTK industry in South Australia is one of the largest producers of cultured YTK in the world, having sold 3757 tonnes in 2022 worth $AUD66 million. However, a major risk to the development of the YTK industry is posed by disease caused by pathogenic bacteria, which has caused mortality in YTK sea cage fish in Australia and in other *Seriola* species globally [2–5]. Additional impediments include the appearance of resistant strains and food safety concerns associated with the use of antibiotics. These factors have driven the aquaculture industry to find safe and effective dietary additives that can strengthen the immune response and improve the welfare of marine cultured fish [6–8].

The use of yeast and products derived from the yeast cell wall, such as mannan oligosaccharides (MOSs), has received a great deal of attention in aquaculture due to their beneficial effects on fish health [9]. The main benefit derives from their direct adhesion to enteropathogenic bacteria and prevention of adhesion to the gut epithelium [10]. This action causes bacteria to be removed with faeces and reduces the incidence and severity of the potential disease [11]. Yeast-derived MOSs have also been reported to act as prebiotics due to the presence of non-digestible low-molecular-weight carbohydrates, which are preferentially fermented by intestinal lactic acid bacteria [12]. This has been shown to positively enhance the gut microflora and mucosal barrier immunity in various aquaculture species [12]. The latter has been further validated by studies which reported the promotion of gut pre-epithelial and epithelial defence mechanisms by enhancing mucus secretion and mucosal barrier function in various fish species when they are fed diets supplemented with MOS [11]. Concomitantly, mucus and goblet cell density in skin were improved by MOS supplementation in Atlantic salmon [12]. MOSs bind with pattern recognition receptors such as the endocytic mannose-receptor, which is essential for both pro- and anti-inflammatory cytokine production [13].

Organic acids have been tested in aquafeed with beneficial effects on farmed fish [14]. Monoglycerides are esters formed by glycerol and one molecule of fatty acid (derived from short-chain fatty acids [SCFAs] and medium-chain fatty acids [MCFAs]) have attracted commercial and research attention for use as an additive in aquafeed [14]. The main benefit of monoglycerides is that intestinal lipases can only break down the glycerol backbone, resulting in the release of organic acids, meaning that SCFA or MCFA are able to reach the distal portion of the intestine without the interference of the upper gastrointestinal tract, allowing the acids to exert their main functions [15, 16]. These functions relate to antimicrobial activity and the proliferation of *Lactobacillus* bacteria, which are beneficial in the hindgut of fish [14]. Moreover, due to their amphipathic properties, monoglycerides can destabilise bacterial membranes by pore formation [14], which leads to cell lysis as a result of increased cell permeability [17]. The efficacy of monoglycerides in promoting growth and antimicrobial activity has been well-documented in poultry [18–21], but their effects on fish have been poorly studied. However, a diet supplemented with 0.5% monoglyceride was reported to enhance the intestinal microbiota by increasing the number of beneficial lactic acid bacteria (specifically *Lactobacillus*) with a simultaneous decrease in the abundance of potentially pathogenic bacteria belonging to Gammaproteobacteria group in gilthead sea bream (*Sparus aurata*) [14].

Polyphenols are one of the most abundant naturally occurring bioactive secondary metabolites produced by plants to defend themselves against bacterial, viral and fungal infection [22], with known biological activities and potential health benefits in aquaculture. The beneficial influences of dietary polyphenols and polyphenol-rich diets on growth performance, antioxidant defences, and immunity in farmed fish have been well reported [23–30]. In particular, polyphenolic components have been found to improve skin mucus immunity and intestinal mucosal health by decreasing proinflammatory cytokines (*IL-1β* and *TNF-α*) expression and by upregulating the *mucin 2* expression in turbot (*Scophthalmus maximus*) [31].

Despite the evidence of the efficacy of yeast and yeast-derived products, monoglycerides and polyphenols on a variety of aquaculture fish species, little evidence is available concerning the efficacy of such additives for YTK health. Stone et al. [32] trialled spent brewer's yeast (BY) as an additive in juvenile YTK diets at 20% inclusion and found no benefits to the specific growth rate (SGR) or feed conversion ratio (FCR) after 70 days. For that reason, the present study was designed to investigate the dietary supplementation of yeast and yeast-derived products, a monoglyceride product and polyphenol product on the growth, skin and gut health and resistance to *Photobacterium damselae* subsp. *damselae* (Pdd) in YTK.

## 2. Materials and Methods

### 2.1. Ethics Statement

All procedures for handling of experimental fish were carried out in strict accordance with the guidelines and regulations in Australia for the care and use of scientific animals and following a review and approval by the Animal Ethics Committee at the Department of Primary Industries and Regional Development (DPIRD), Western Australia (Permit Number: AEC-20-3-07). A recommended dose of anaesthesia and euthanasia (Aqui-S) was used as per the standard operating procedure (SOP) of the Marine Fish Hatchery, DPIRD to minimise pain and discomfort during sampling and culling.

### 2.2. Experimental Diets

The dietary supplements were supplied by Ridley Agriproducts, and due to commercial sensitivities, the trade names are excluded. All dietary supplements were applied to a commercial diet (Pelagica, Ridley Agriproducts, Queensland, Australia) at either the manufacturer's recommended rate or as prescribed by Ridley Agriproducts (pers. comm., R. Smullen; Table 1). Products were top-coated to the diet using 50 mL·kg^−1^ of a 20% w/v gelatine solution according to Partridge et al. [33]. Due to commercial-in-confidence agreements with the manufacturers, the detailed chemical composition of the tested additives, including the concentrations of active ingredients, such as polyphenols, monoglycerides, MOSs and superoxide dismutase (SOD), was not disclosed. As such, the inclusion levels reported in this study are based on the commercial product as supplied, without further analytical confirmation of active compound concentrations.

### 2.3. Growth Trial and Sampling

Juvenile YTK were sourced from the DPIRD commercial nursery (Perth, Australia). Before the commencement of trial, 336 fish (50.0 ± 0.1 g) were individually weighed, microchipped and transferred into 24 500 L tanks (8 treatments in triplicate, 14 fish·tank^−1^). The tanks were supplied with ambient temperature (20 ± 1°C) oxygenated seawater at a rate of 6 L·min^−1^. Fish were fed to satiety twice daily, and food intake was recorded for 55 days. On day 28, fish were fasted 24 h prior to anaesthesia and individually weighed each fish to determine weight gain and SGR. On day 28, three fish/treatment were stripped for faecal sampling for myeloperoxidase activity (MPO) analysis. These fish were then removed from the nutrition study, and their body weights were recorded to calculate feed conversion efficiency. These fish were recovered in a separate experiment facility for 4 days before performing the non-lethal immersion challenge trial as described below. All fish in the faecal-stripped cohort were visually observed consuming their respective feed during the 4-day post-stripping period and again following the bath challenge, prior to faecal collection.

At the end of the 55-day feeding trial, the remaining 11 fish were individually weighed to determine the final growth performance. Three fish per tank were euthanised, 24 h after final feeding, with Aqui-S (40 mg·L^−1^), followed by cutting of the cervical spine, the left and right flanks were scraped for mucus, and the skin mucus quantity was calculated, and the liver was dissected and weighed to calculate hepatosomatic index (HSI). The following equations were applied:

  $$\begin{array}{c} \text{Weight gain }\left(g\cdot {\text{fish}}^{-1}\right)=\text{final weight }-\text{initial weight}, \end{array}$$

  $$\begin{array}{c} \text{SGR }\left(\%\right)=\left(\frac{\ln\text{ final weight }-\ln\text{ initial weight}}{\text{Days}}\right)\times 100, \end{array}$$

  $$\begin{array}{c} \text{FCR }=\frac{\text{Biomass gain }\left(g\right)}{\text{Total feed consumed }\left(g\right)}, \end{array}$$

  $$\begin{array}{c} \text{HSI }\left(\%\right)=\left(\frac{\text{Liver weight }\left(g\right)}{\text{Body weight }\left(g\right)}\right)\times 100, \end{array}$$

  $$\begin{array}{c} \text{Skin mucus }\left(\text{mg}\cdot {\text{mm}}^{-1}\right)=\frac{\text{Skin mucus weight }\left(\text{mg}\right)}{\text{Fork length }\left(\text{mm}\right)}. \end{array}$$

Of the remaining 24 fish/treatment, 20 fish/treatment were subjected to an IP challenge trial, as described below, and the remaining 4 fish/treatment (a total of 32 fish) were culled humanely.

### 2.4. Histological Analysis of Liver, Skin and Hindgut

Samples of the posterior hindgut, skin, spleen and liver were dissected from each of the three sampled fish and fixed in 10% neutral buffered formalin. Following tissue dehydration, clearing in xylene, embedding in paraffin and sectioning with a microtome, sections were stained in either haematoxylin and eosin (H&E) or periodic acid-Schiff and Alcian Blue slides [34, 35]. Slides were scanned at 40x magnification using an automated slide scanning system (Olympus VS200 Slide Scanner, Olympus Corporation, Japan) and examined by imaging software (Olympus VS200, Olympus Corporation). Hindgut mucosal morphology, including the lengths of the muscularis and submucosa thickness, total villi length, villus and lamina propria width and total number of mucous cell counts (cells per 100 µm/2) was performed. Epidermal measurements included epidermis thickness and mucous cell counts (cells per 100 µm).

### 2.5. Gene Expression Analysis

The relative expression of the reference gene (elongation factor-1α, *EF-1α*) [2], inflammatory cytokines (tumour necrosis factor alpha, *TNF-α*; interleukin 10, *IL-10* and interleukin-8, *IL-8*), mucin production relevant genes (*mucin 2* and i*-mucin*), stress relevant gene (*Hsp70*), antioxidative genes (*CAT* and *SOD*) and mucins synthesis relevant genes (*calretuculin*) were quantified by quantitative real time PCR (qPCR) (Table 2). Total RNA was extracted from 30 mg of hindgut and skin tissue that was stabilised in RNAlater. Tissues were disrupted in 600 µL of RLT buffer using a homogeniser (Precelleys Evolution, Bertin Technologies). RNA was extracted using a RNeasy Mini kit (Cat #74106, Qiagen, Australia) following the manufacturer's instructions. The RNA quantity and purity were measured by Nanodrop spectrometry (ND-1000, Thermo Scientific). Complementary DNA (cDNA) was synthesised by reverse transcription reaction using QuantiTect Reverse Transcription kit (Cat #205311, Qiagen, Australia) with 1 µg of total RNA and included a genomic DNA elimination step. The qPCR was performed using a Rotor-gene Q Thermocycler (Qiagen, Germany) in a 72-rotor disc in triplicate 25 µL reactions. Each reaction contained 12.5 µL of SensiMix SYBR NO-ROX (Cat # QT650-05, Bioline, Australia), 0.5 µL of forward and reverse primers, 8.5 µL of RNase-free water and 3 µL of cDNA. Each qPCR was carried out with a negative control (non-template control) containing no cDNA; thermocycling conditions had an initial denaturing step at 95°C for 10 min, followed by 40 cycles of 95°C for 30 s, 60°C for 30 s and 72°C for 30 s. Amplification was followed by a standard melt curve from 72 to 95°C, in increments of 1°C for 5 s at each step to confirm that only one product was amplified. All genes were not amplified with both tissue types: *IL-8*, *CAT* and *calreticulin* amplified with skin tissue and *IL-10* and *i-mucin* amplified with hindgut tissue (Table 2). Samples were run in parallel with the reference gene. The primer efficiency for all primers was assessed to ensure optimised and reproducible assays. The change in gene expression was reported as ΔΔCt.

### 2.6. MPO

At day 28, three fish per tank were stripped following the techniques described by Pilmer et al. [36] and Dam et al. [37] to collect faecal samples for quantification of MPO as an indicator of hindgut inflammation. Briefly, a gentle pressure was applied to the abdomen of anaesthetised fish to push out urinary products. The ventral area was wiped, and gentle abdominal pressure was applied again to collect faecal material into a clean container. MPO activity of the faecal samples was analysed following the protocols as illustrated in Woolley et al. [38]. Briefly, a solution of 0.5% CTAB buffer (hexadecyltrimethylammonium bromide)/50 mM phosphate-buffered saline (PBS) buffer was used to homogenise snap-frozen (−80°C) faecal samples, which were centrifuged, and the supernatant removed. Human MPO (Cayman Chemical, Australia) was used to prepare the standards and then diluted in CTAB PBS buffer. Experimental samples and MPO standards were pipetted into a black 384-well microplate in triplicate. A working solution of 10 μM of benzoic acid (APF, Cayman Chemical, Australia) and 10 μM hydrogen peroxide in PBS was then added into each microplate well. The microplate was incubated in the dark for 30 min at room temperature, and fluorescence was measured using excitation (485 nm) and emission (515–530 nm) wavelengths every minute on a fluorescence plate reader (Clariostar, BMG, Germany). Sample protein quantification was performed with a detergent-compatible Protein Assay (Bio-Rad, Australia) and read at 750 nm using a BioTek Powerwave XS Spectrophotometer with KC4 (v 3.4) programme. The results were demonstrated as nmol·mg^−1^ of protein.

### 2.7. Bacterial Challenge

Those fish that were faecal stripped on day 28 were recovered in a separate system and fed their treatment diets for 4 days before being transferred to the bacterial challenge facility. The fish were transferred into 24 × 300 L tanks, the water level in each tank was lowered to 50 L, and Pdd bacterial broth was administered to each tank at a non-lethal dose of 1 × 10^6^ CFU·mL^−1^. After 1 h, water flow was restored to 8 L·min^−1^. Fish were fed on their respective treatment diets immediately post-challenge. Eighteen hours post-challenge, fish were individually stripped for faecal samples following the method described above. The concentration of Pdd in the faeces was quantified by diluting 100 mg of faecal matter in 1 mL of PBS and serially diluting from 10^1^ to 10^4^, 100 µL of diluents 10^2^ and 10^4^ were lawn plated on marine agar and incubated at 25°C for 48 h, and the colonies were counted.

On day 55, 20 fish per treatment were transferred to the challenge facility, where they were challenged with an IP injection of Pdd at 2.4 × 10^5^ CFU·mL^−1^ in a mixed model challenge trial (20 replicate tanks with 1 fish per dietary treatment in each tank). The bacterial strain used was a plasmid-bearing strain described by Gupta et al. [39]. The inoculation was administered through an IP injection of 0.1 mL of broth. The broth was prepared by lawning 100 µL of a stock defrosted from −80°C onto a blood agar plate, then incubating for 24 h at 24°C. Following this, two 1 µL loops were taken from this plate and placed in 1 mL of PBS. 100 µL of this solution was then lawned onto another blood agar plate and incubated for 24 h at 24°C. The bacteria were harvested from the plate by rubbing the colonies off using a sterile cotton swab and rinsing into 5 mL sterile PBS. The concentration of the solution was assumed to be 10^9^ CFU·mL^−1^ and was confirmed by serial dilution plate counts. The final challenge broths were then prepared using sterile PBS, and their concentrations were checked using the Bactiquant fluorometer and serial dilution plate counts both immediately before and after challenge to confirm the challenge dose. As soon as fish displayed any sign of infection (e.g., lesions, darkness of colour, abnormal or erratic swimming), they were euthanised using Aqui-S (40 mg·L^−1^), followed by cutting of the cervical spine. Any such infected fish were considered to be mortalities and used to calculate the survival rate.

### 2.8. Statistical Analysis

Results are reported as mean ± standard error (SE). All data were subjected to one-way ANOVA, unless stated otherwise, to determine the effect of dietary treatment. When the effect was significant, results were compared among the diets by Tukey HSD using JMP software (version 14, SAS Institute Inc., Lane Cove, Australia). A chi-square test for independence was conducted to determine the relationship between dietary treatments and the presence of Pdd in the faeces following the first, non-lethal challenge trial. Survival data obtained from the second challenge trial were analysed by the Kaplan–Meier method using the log-rank (Mantel–Cox) test to compare test diets against the control. Data were normalised by arcsine transformation prior to analysis if needed. The level of significance was set at *p* < 0.05.

## 3. Results

### 3.1. Growth, Feed Intake and Survival

There was no mortality in any dietary treatment during the 55-day growth trial. Weight gain over the 55-day trial ranged from 207 ± 4 g in the M-low treatment to 238 ± 10 g in the control diet; however, the differences between treatments were not significant (*p* = 0.18) (Table 3). Food intake was negatively impacted by treatment (*p* = 0.04), with fish offered the M-low treatment consuming significantly less feed (2.84 ± 0.01 kg) than those fish fed the control diet (3.25 ± 0.05 kg). FCR ranged from 1.11 ± 0.03 (control and M-low) to 1.19 ± 0.02 (P-low) and was affected by treatment (*p* = 0.02), with the P-low treatment resulting in significantly higher (worse) FCR than the control. HSI was similar across the treatments (*p* = 0.56).

### 3.2. Liver and Spleen Health

**Figure 1:**
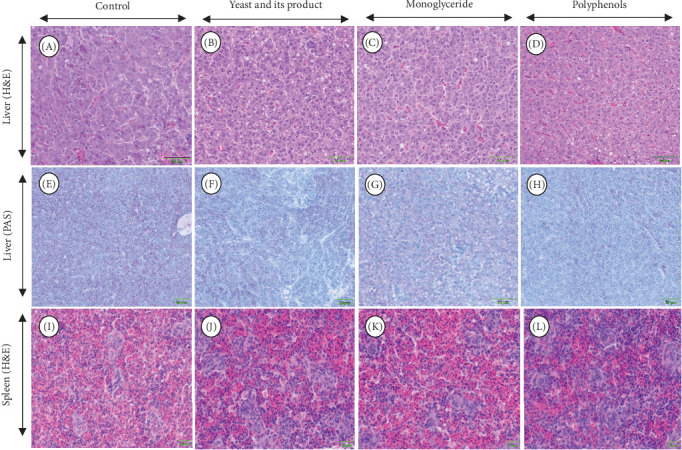
(A–D) The representative liver micrograph stained with H&E, (E–H) PAS and (I–L) spleen (H&E) of YTK fed control, and diets containing various additives, including yeast and yeast-derived product, monoglyceride and polyphenols after 55 days.

Histological analysis demonstrated that the dietary additives tested in this study did not cause any pathological changes in the liver microstructure, as evidenced by the regular appearance of polygonal hepatocytes with a distinctive central nucleus with densely staining chromatin margins and exocrine pancreas with zymogen (Figure 1A–D). Periodic acid-Schiff stain was used to detect glycogen deposits in the hepatocytes from liver tissue sections, as demonstrated in Figure 1. Periodic acid-Schiff positive cells indicated an adequate amount of glycogen storage in the cytoplasm in all fish, regardless of dietary treatment (Figure 1E–H). Likewise, spleen microstructure was normal in YTK fed all diets, supported by adequate haematopoietic tissue and no observed increase in numbers of melanomacrophage centres or haemosiderin (Figure 1I–L).

### 3.3. Skin Mucosal Barriers

**Figure 2:**
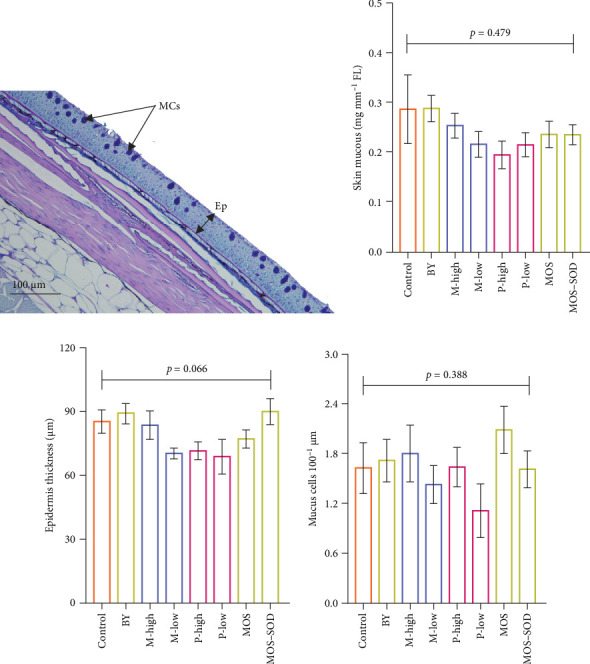
(A) The representative skin micrograph, (B) skin mucus quantity (mg mucus·mm^−1^), (C) epidermis thickness (Ep) and (D) mucus cells (MCs) of YTK fed diets containing various additives, including yeast and yeast-derived product, monoglyceride and polyphenols after 55 days.

Mucus quantity demonstrated no significant differences among treatments (*p* = 0.49) (Figure 2B). Similarly, quantitative skin histological measurements, as demonstrated in Figure 2A, found that skin epidermis thickness (*p* = 0.06; Figure 2C) and number of mucus cells (*p* = 0.38; Figure 2D) were unaffected by the supplementation of various additives.

### 3.4. Immune-Relevant Genes Expression in Skin

**Figure 3:**
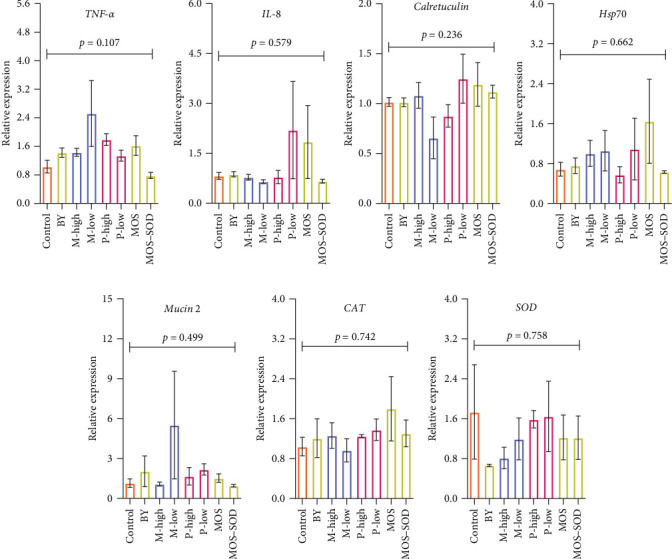
(A, B) Pro-inflammatory genes (*TNF-α* and *IL-8*), (C) calreticulin, (D) stress related gene (*HSP70*), (E) mucin production relevant genes (*mucin 2*) and (F, G) antioxidative gene (*CAT* and *SOD*) in the skin of YTK fed diets containing various additives, including yeast and yeast-derived product, monoglyceride and polyphenols after 55 days.

The fold change in expression of pro-inflammatory genes (*TNF-α* and *IL-8*), stress-related gene (*Hsp70*), mucin synthesis-relevant gene (calreticulin), mucin production-relevant genes (*mucin 2*) and antioxidative gene (*CAT* and *SOD*) in the hindgut of YTK in response to various additives are presented in Figure 3. There were no significant effects of additive supplementation on the expression of the above-mentioned genes (*p* > 0.05; Figure 3A–E).

### 3.5. Hindgut Mucosal Barriers

**Figure 4:**
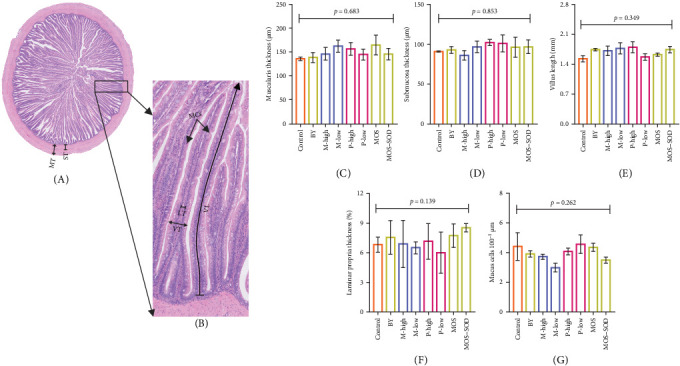
(A, B) The representative hindgut micrograph and the quantitative measurements, including (C) muscularis thickness (MT), (D) submucosa thickness (ST), (E) villus length (VL), (F) percent laminar propria thickness and (G) mucus cells (MCs) of YTK (*n* = 3) fed diets containing various additives, including yeast and yeast-derived product, monoglyceride and polyphenols after 55 days.

The quantitative measurements of hindgut histomorphology are presented in Figure 4, including villus length (Figure 4B), laminar propria thickness (Figure 4C), number of mucus cells (Figure 4D), muscularis thickness (Figure 4E) and submucosa thickness (Figure 4F). None of these histomorphological metrics was affected by the inclusion of the additives.

### 3.6. Immune-Relevant Genes Expression in Hindgut

**Figure 5:**
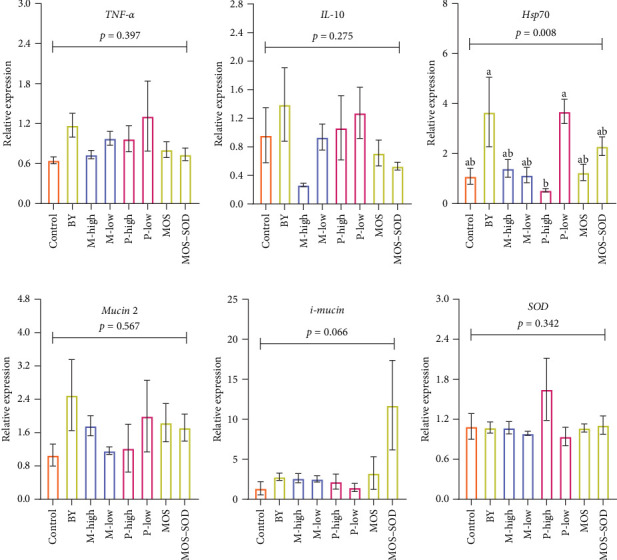
(A, B) Inflammatory genes (*TNF-α* and *IL-10*), (C) stress-related gene (*Hsp70*), (D, E) mucin production relevant genes (*mucin 2* and *i-mucin*) and (F) antioxidative gene (*SOD*) in the hindgut of YTK (*n* = 3) fed diets containing various additives, including yeast and yeast-derived product, monoglyceride and polyphenols after 55 days. Bars with different letters represent significant differences between treatments.

The fold change in the expression of inflammatory genes (*TNF-α* and *IL-10*), stress-related gene (*Hsp70*), mucin production-relevant genes (*mucin 2* and *i-mucin*) and antioxidative gene (*SOD*) in the hindgut of YTK, in response to various additives, is presented in Figure 5. Though both *TNF-α* (Figure 5A) and *IL-10* (Figure 5B) showed an increased fold change in the hindgut of YTK fed P-low, none differed statistically when compared with those fish fed the control diet. YTK fed BY and P-low demonstarted an increase in the fold change of *Hsp70* compared to those fed P-low (*p* = 0.008; Figure 5C); however, there was no variation between control and treatment diets. Mucin production relevant genes, including *mucin 2* (Figure 5D) and *i-mucin* (Figure 5E) were not influenced by additive supplementation; however, an elevation was found in YTK-fed BY and MOS–SOD when compared with control. The antioxidative gene, *SOD* (Figure 5F) showed a similar response to other genes.

### 3.7. MPO

**Figure 6:**
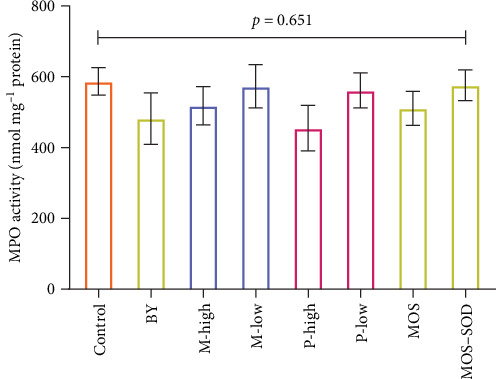
Myeloperoxidase activity (mean ± SE) in the faecal matter of YTK fed diets containing various additives, including yeast, a yeast-derived product, monoglyceride and polyphenols over 28 days.

Supplementation of the various additives did not influence the faecal matter MPO activity (*p* = 0.651; Figure 6).

### 3.8. Bacterial Challenges

**Figure 7:**
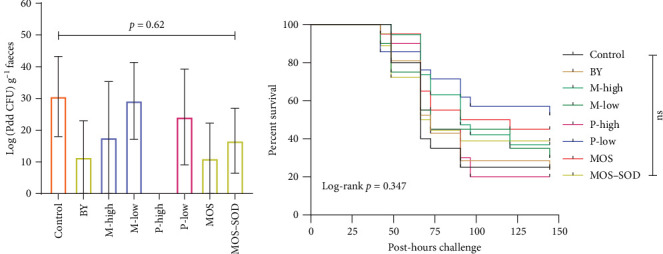
(A) The effect of dietary additives on faecal Pdd excretion in challenged YTK and (B) the survival of YTK post-challenge with Pdd at 2.4 × 10^5^ CFU·mL^−1^ fed diets containing various additives, including yeast, a yeast-derived product, monoglyceride and polyphenol. “ns” indicates no significant different between the treatments (*p* > 0.05).

Following the first bath challenge, additives were found to have no significant effect on the concentration of Pdd in the faeces (*p* = 0.62; Figure 7A). The degree of error within treatments was very high, with some treatments having four out of the five replicates containing no Pdd; however, there was no significant difference between the treatment groups and the presence of Pdd in the faeces (*χ*^2^ = 7.18; *p* = 0.410). Despite the lack of difference amongst treatments, those fish fed the high inclusion of polyphenol product were found to have no Pdd within the faeces across all replicates.

The survival of fish fed the control diet following Pdd infection was 25% (Figure 7B). Survival of fish in most other treatments was higher than the control, ranging from 54% (P-low) to 30% (M-low), however, none of the dietary treatments resulted in statistically significant differences relative to the control (*p* = 0.347).

## 4. Discussion

There has been increasing interest in the use of functional ingredients with immuno-modulating properties in aquafeeds for assisting in disease-resistant and chemotherapeutic-free sustainable aquaculture. This study evaluated several immunostimulants, including BY, yeast and yeast-derived products, a monoglyceride and a polyphenol product, to investigate their potential benefits on YTK health.

Numerous studies have reported the beneficial effects of yeast and yeast-derived products on the growth performance of fish [40]. However, in the present study, no such effects were found when YTK were fed BY at 2.0 g·kg^−1^ and yeast-derived products at 2.0 and 0.9 g·kg^−1^ for 55 days. This is consistent with [32], who found no beneficial effects of YTK from adding spent BY at the much higher inclusion level of 20% to fishmeal and soybean meal-based diets. Similarly, feeding greater amberjack (*Seriola dumerili*) juveniles for 90 days with diets supplemented with commercial yeast-derived products, Bio-Mos (5 g·kg^−1^) or Actigen (2 g·kg^−1^) or their combination, did not influence growth nor feed efficiency [41, 42]. The findings in the present study and previous *Seriola* species suggest there is no benefit to the feed utilisation or growth of juvenile YTK by adding yeast and yeast-derived products to feeds.

In the same vein, supplementation with the polyphenol product had no influence on YTK growth at either of the inclusion levels tested (6 and 12 g·kg^−1^), although this was in contrast to several studies that found positive effects of polyphenols and polyphenol-rich additives on the growth performance of various finfish [43] such as olive flounder (*Paralichthys olivaceus*) [44], beluga sturgeon, (*Huso huso*) [29] and turbot [31]. Similarly, monoglyceride supplementation did not improve the growth performance of YTK in this study, either at low levels or high levels. However, the significant reduction in feed intake observed in fish fed M-low suggests that prolonged feeding would likely have led to reduced growth. These differences from previous studies indicate that the effects of immunostimulants on growth performance are highly dependent on both dosage and fish species.

Quantifying intestinal histomorphology aids the understanding of the potential influence of immunostimulants on intestinal mucosal functions and overall health of fish. The present study found no positive effects of yeast or yeast-derived products on YTK intestinal mucosal barrier function, as quantified by villus length, lamina propria, mucus cells, muscularis thickness and submucosa thickness. These results contradict other studies that found positive effects of yeast-derived products on intestinal mucosal barrier functions in fish, including other marine carnivorous species [11, 12, 45]. Such discrepancies could be attributed to different species, product structures, inclusion levels and supplementation durations.

Similarly, low and high inclusions of polyphenols had no effect on the intestinal mucosal barriers of YTK. In contrast, polyphenols derived from tea plants have been shown to improve gut mucosal barrier in spotted sea bass (*Lateolabrax maculatus*) by increasing the height of the intestinal villus [46], suggesting that fish gut health responses likely depend on the sources of polyphenols or the fish species to which they are being fed. Likewise, low and high monoglyceride supplementation did not influence YTK intestinal mucosal barrier function. The lack of histomorphological changes in the hindgut mucosal barrier function aligned with results of the expression of hindgut inflammatory cytokines (*TNF-α* and *IL-10*), stress-relevant gene (*Hsp70*), mucin production-relevant genes (*mucin 2* and *i-mucin*) and antioxidative gene (*SOD*).

The equilibrium between pro-inflammatory cytokines (e.g., *TNF-α*) and anti-inflammatory cytokines (e.g., *IL-10*) is crucial for immune response homeostasis and inflammation. Increased expression of *TNF-α* with a concurrent decrease in *IL-10* expression has been associated with intestinal inflammation in fish. Feeding European seabass (*Dicentrachus labrax*) a diet supplemented with a multi-strain yeast fraction (0.8 g·kg^−1^) preserved a healthy intestinal mucosal barrier by upregulating the expression of the pro-inflammatory cytokine *IL-1β* and the anti-inflammatory cytokine *IL-10* [45]. In the present study, neither BY, yeast-derived products, nor low and high monoglyceride additives resulted in statistically significant changes in the expression levels of *TNF-α* and *IL-10* in the hindgut of YTK. While non-significant trends were observed in the P-low and BY treatments, these were not consistent or robust enough to suggest definitive immune modulation. Similarly, a trend toward reduced *IL-10* expression in M-high was observed, but again, lacked statistical significance. As such, while the expression profiles hint at potential differential responses to some additives, the variability within treatments and the lack of significant effects preclude drawing conclusions about the relative advantages of one product over another.

While YTK fed a low level of polyphenol (6 g·kg^−1^) showed a trend toward higher survival and upregulated expression of *TNF-α* and *IL-10* in the hindgut compared to the control, these differences were not statistically significant. This trend was consistent with higher post-challenge survival observed in the P-low fed fish compared to the control treatment. Similarly, supplementation with 1, 5 and 10 g·kg^−1^ of apple polyphenols attenuated low-fishmeal-induced intestinal inflammatory response in grass carp (*Ctenopharyngodon idellus*) by controlling the expression levels of pro-inflammatory cytokine genes such as *IL-1β*, *IL-6* and *TNF-α* [47]. However, fish fed the high polyphenol dose (12 g·kg^−1^) exhibited lower survival than the control group following systemic (IP) Pdd infection, despite the absence of Pdd in faeces after the initial mucosal (bathe) challenge. This suggests that while some mucosal immune interaction may have occurred, it did not translate into enhanced systemic protection. These results indicate that further dose optimisation is required before drawing conclusions about the efficacy of polyphenol supplementation

Supplementation with a single-strain yeast fraction or multi-strain yeast fraction was reported to downregulate the expression of intestinal *Hsp70*, a stress response gene induced during exposure to various stressors in aquatic organisms, in European seabass [45] and rainbow trout (*Oncorhynchus mykiss*) [48]. Hindgut expression of *Hsp70* was significantly higher in fish fed BY and the low-dose polyphenol (P-low) diet compared to the high-dose polyphenol (P-high) group (*p* = 0.008). However, none of the additive treatments differed significantly from the control diet. Hsp70 is a stress-responsive gene often upregulated in response to environmental or physiological stress. The increase observed in the BY and P-low groups may suggest a mild stress response or cellular adaptation to the dietary treatment. However, since no consistent stress markers were observed across other genes or tissue types, the biological relevance of this isolated upregulation remains unclear and warrants further investigation. The lowest *Hsp70* expression in YTK fed P-high compared to other additives suggested the efficacy of polyphenols at 12 g·kg^−1^ on reducing the stress of YTK. This may partly explain the absence of Pdd in faeces of the same dietary group in response to the non-lethal immersion challenge trial. Similarly, tea polyphenols at very low supplementation levels (0.4–1.6 g·kg^−1^) alleviated high lipid diet-induced stress by controlling the expression of *Hsp70* and *Hsp90* in the liver of juvenile hybrid grouper (*Epinephelus fuscoguttatus* ♀ × *E. lanceolatus* ♂) [49]. However, both *mucin 2* and *i-mucin* in hindgut were unchanged by additive supplementation, which was consistent with skin mucin 2 levels.

Polyphenols are known for their ability to regulate antioxidant responses in fish. Yang et al. [47] found an elevation in intestinal CAT and SOD activity, with concurrent upregulation of *CAT* and *CuZnSOD* genes in grass carp when fed 5 and 10 g·kg^−1^ of apple-derived polyphenols. Similarly, supplementation with 0.01%–0.06% tea polyphenols improved antioxidant capacity in the serum (CAT, GSH-Px, T-AOC) and liver (SOD, CAT, GSH-Px, T-AOC) by upregulating the expression of *CAT* and *SOD* in juvenile hybrid grouper (*Epinephelus fuscoguttatus* ♀ × *E. lanceolatus* ♂) [49]. However, in this present study, such beneficial effects were not observed with the polyphenol product used, as evidenced by the unchanged expression levels of *SOD* in YTK fed polyphenols.

Leclercq et al. [12] found that a diet containing a single strain of yeast (4 g·kg^−1^) fed to Atlantic salmon significantly improved skin mucus production (+46%) and goblet cell density (+25%) at the end of 6-week trial. However, the yeast and yeast-derived products used in this study did not enhance skin mucosal protection in YTK after a similar duration, with no observed changes in mucus quantity, epidermis thickness, or mucus cells in the skin. Similarly, supplementation with the monoglyceride and polyphenol products did not improve skin mucosal function. The lack of response in skin mucosal functions was consistent with hindgut mucosal functions, with both mucosal responses aligning with the results of inflammatory cytokines and mucin-relevant gene expression in the hindgut and skin of YTK.

The skin inflammatory cytokine response, specifically *TNF-α* and *IL-8* expression, was unchanged by supplementation with yeast, yeast-derived products, monoglyceride product, or polyphenol product. In contrast, the expression of *TNF-α* and *IL-8* significantly increased in the skin of greater amberjack fed 2.0 g·kg^−1^ MOS, a yeast-derived product, for 90 days [41]. *Calreticulin*, a multifunctional gene in fish mainly associated with the synthesis of mucins, has been reported to be stimulated in Atlantic salmon [50], rainbow trout and European seabass [51] when fed yeast-derived products. In this study, fish fed the yeast-derived product and a low inclusion of polyphenol tended to have higher expression of *calreticulin*, though the variation in comparison to the control diet was not statistically significant. Similarly, none of the additives influenced the expression of *Hsp70*, *mucin 2*, *CAT* and *SOD* in the skin of YTK, which was consistent with the expression of these genes in the hindgut of YTK. Taken together, these findings suggest that yeast, yeast-derived products, monoglyceride product and polyphenol product were unable to stimulate mucosal-associated lymphoid tissue, particularly regarding intestine and skin health.

Yeast and yeast-derived products have been well studied as immunostimulants due to their ability to enhance disease resistance by stimulating the immune system of fish. Oral administration of insoluble yeast β-glucans significantly reduced the load of pathogenic *Vibrio* genus in the gut of Senegalese sole (*Solea senegalensis*) [52]. Mortality was lower in YTK fed the MOS (55%) and the combination of MOS and SOD (61%) diets relative to those fish fed the control (75%); however, the differences were not significant. A similar finding was made by Torrecillas et al. [53] in European sea bass, where a diet supplemented with MOS at 4 g·kg^−1^ reduced the mortality (13%) against *V. anguillarum* exposure than those fed the control diet (67%). Faecal excretion of Pdd was lower in post-challenge fish fed the yeast products (BY, MOS and MOS–SOD) compared to fish fed the control diet. Similarly, piglets had a significantly decreased *Escherichia coli* in their faeces when fed a blend of inactivated yeast strains (equivalent to the MOS product used in this study) over 22 days [54].

The antimicrobial properties of polyphenols have previously been proven by an in vitro study [55]. This was underpinned by Van Doan et al. [56] and Bin Ma et al. [57], who found improved immunity in Nile tilapia (*Oreochromis niloticus*) against *Streptococcus agalactiae* and grass carp against *Aeromonas hydrophila* when fed polyphenol-supplemented diets. Though statistical differences in survival were not observed in the present study, where YTK were fed either 6 or 12 g·kg^−1^ polyphenol inclusion diets, the mortality rate was much lower in YTK fed 6 g·kg^−1^ polyphenol (48%) when compared to the fish fed the control (75%).

Moreover, the role of active ingredients in polyphenols is often unpredictable and not specific to bacterial species [58]. For instance, an earlier study tested the efficacy of four phenolic compounds, including pyrogallol, syringic, rutin and vanillic acid on *V. parahaemolyticus* and found only the pyrogallol compound to have any strong antibacterial activity [59]. This may explain the lack of consistent or statistically significant beneficial effects of polyphenol supplementation against Pdd in this study, despite suggestive trends in faecal shedding and survival outcomes at the lower dose. The absence of Pdd in the faeces of fish fed the P-high diet may indicate localised mucosal protection or rapid pathogen clearance in the gut. However, given the small sample size (*n* = 3 fish per tank), caution is warranted in interpretation. Moreover, the IP challenge bypasses mucosal barriers and elicits systemic immune responses, which may explain why P-high fish did not show improved survival, despite apparent mucosal protection. Similarly, YTK fed the monoglyceride product found no immunostimulatory effects. The non-immunostimulatory effects of various additives against Pdd were further validated by hindgut MPO activity, a specific haemoprotein released by neutrophils to provide antimicrobial protection [60, 61].

A limitation of this study is the lack of detailed compositional data for the commercial additives due to confidentiality agreements. Without knowing the exact concentrations of active compounds in the tested additives, it is difficult to fully interpret the mechanisms behind the observed effects. Future studies should include chemical analyses of additives to improve clarity, reproducibility and cross-study comparisons.

## 5. Conclusion

**Figure 8:**
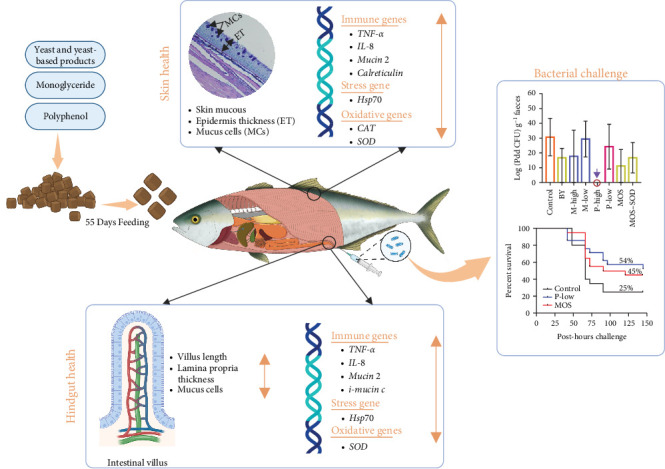
Schematic diagram summarising the overall responses of YTK to dietary supplementation with various additives, including yeast and yeast-derived products, monoglycerides and polyphenols.

Despite the well-documented benefits of yeast and yeast-derived products, monoglycerides and polyphenols on the growth performance of other fish species, no such effects were observed in YTK in the present study. These findings suggest that the effects of immunostimulants on fish health and growth are highly species-specific and dose-dependent. The inability of the tested supplements to stimulate mucosal-associated lymphoid tissues indicates that the functional benefits observed in other fish species do not necessarily apply to YTK under the conditions tested in this study. The lack of significant immunostimulatory effects was also consistent with crucial genes expression related to inflammatory cytokines (*TNF-α*, *IL-10 and IL-8*), stress response (*Hsp70*), mucin synthesis and production (*mucin 2*, *i-mucin* and *Calreticulin*), or antioxidative response (*CAT* and *SOD*) in either the hindgut or skin of YTK (Figure 8). However, when fish were challenged with a bacterial pathogen, the fish fed diets supplemented with 0.8 g·kg^−1^ of MOS or 6 g·kg^−1^ polyphenol had higher survival outcomes, when compared to those fish fed the control (Figure 8). The immunostimulating capacity of polyphenols was further proven by the absence of Pdd in the faeces of YTK fed the polyphenol high-dose treatment. The results suggest there may be merit in further studies of MOS and polyphenol products in dietary supplementation for YTK. Future studies should also prioritise full chemical characterisation of feed additives to better elucidate their modes of action and support standardised applications in aquaculture.

## Figures and Tables

**Table 1:** Dietary supplements and their concentration fed to juvenile YTK over 55 days.

Product	Dose (g·kg^−1^)	Code
Control, no additive	—	Control
Brewer's yeast	2.0	BY
Mannan-oligosaccharide	0.8	MOS
Mannan-oligosaccharide + superoxide dismutase	0.8 + 0.1	MOS–SOD
Monoglyceride	5.0	M-low
Monoglyceride	7.5	M-high
Polyphenol	6	P-low
Polyphenol	12	P-high

**Table 2:** List of primers used for gene expression analysis in the hindgut and skin of YTK fed various additives over a period of 55 days.

Target gene	Primer sequence	Tm (°C)	Product size (bp)	Primer efficiency	Accession number
*TNF-α*	F: GCCATTTATCTGGGTGCAGT	58	63	2.1^H,S^	XM_023407137
	R: GTTGGTTTCCGTCCACAGTT	59			
*IL-10*	F: GAAACGACGGAGGACATCA	57	62	2.1^H^	XM_023423737.1
	R: TCGAAGATCTGCTGGATGG	57			
IL-8	F: CAACTTGAGCGAGATGTGGA	58	163	2.0^S^	LC010972
	R: TTCATCTCTGAACTCGGTGCT	59			
*mucin 2*	F: CACCTGTGACCAGATGTTGC	59	65	2.1^H,S^	XM_023422497
	R: GTTCAGGGTCAATCAGTTTGG	57			
*i-mucin*	F: TGTGCTCCTGGTTCGACTC	58	65	2.0^H^	XM_023429625
	R: ACGGTGCAGGAGTACTTGAAA	48			
*Hsp70*	F: AGAAAGTGTGCAACCCCATC	58	63	2.1^H,S^	XM_023395815.1
	R: CACCGTTACCTTCAGGCATT	58			
*CAT*	F: GACTGATCAAGGAATAAAGAATCTGTC	58	74	2.0^S^	XM_023400315.1
	R: TTGCATAATCTGGGTTGGTG	56			
*SOD*	F: GCTCCTTCCAGAAAATGAAAGAG	58	95	2.0^H,S^	XM_023400442.1
	R: TCCACTCTGCTTGTCATAGCC	60			
*Calreticulin*	F: CGAGGACATGGATGGAGAGT	59	88	2.1^S^	XM_023428862.1
	R: AGGGTTGTCAATCTGCTTGG	58			
*EF-1a*	F: GGCCAGATCAATGAGGGTTA	57	179	2.0^H,S^	LC010968
	R: ATGGGCTTCTGTGGAATGAG	58			

*Note:* Primer efficiency for the hindgut (H) and/or skin (S), not all the genes worked for both sample types.

**Table 3:** Growth performance, feed utilisation and body indices of juvenile YTK fed various additives, including yeast, a yeast-derived product, monoglyceride and polyphenols over the period of 55 days.

Performance parameters	Control	BY	M-high	M-low	P-high	P-low	MOS	MOS–SOD	ANOVA-*p*
IW (g)	50 ± 0.2	50 ± 0.2	50 ± 0.2	50 ± 0.2	50 ± 0.0	50 ± 0.2	50 ± 0.1	50 ± 0.1	—
FBW (g)	288 ± 10	287 ± 6	266 ± 20	257 ± 5	266 ± 7	271 ± 6	277 ± 12	275 ± 5	0.191
WG (g)	238 ± 10	237 ± 6	216 ± 11	207 ± 4	215 ± 7	220 ± 6	227 ± 12	225 ± 5	0.182
SGR (%)	3.18 ± 0.06	3.16 ± 0.03	3.03 ± 0.08	2.97 ± 0.03	3.02 ± 0.05	3.06 ± 0.04	3.11 ± 0.08	3.09 ± 0.03	0.150
FI (kg)	3.25 ± 0.05^a^	3.26 ± 0.06^a^	3.23 ± 0.03^a,b^	2.84 ± 0.01^b^	2.99 ± 0.07^a,b^	3.22 ± 0.07^a^	3.16 ± 0.14^a^	3.23 ± 0.03^a^	**0.045**
FCR	1.11 ± 0.03^b^	1.12 ± 0.02^ab^	1.16 ± 0.02^a,b^	1.11 ± 0.02^b^	1.13 ± 0.00^a,b^	1.19 ± 0.02^a^	1.13 ± 0.00^a,b^	1.16 ± 0.00^a,b^	**0.017**
HSI (%)	0.75 ± 0.05	0.85 ± 0.04	0.84 ± 0.05	0.82 ± 0.06	0.80 ± 0.03	0.85 ± 0.06	0.77 ± 0.05	0.93 ± 0.06	0.560

*Note*: Different superscript letters indicate significant difference between the treatments. Bold *p* values indicate significant differenence at *p* < 0.05.

Abbreviations: FBW, final body weight; FCR, feed conversion ratio; FI, feed intake; HSI, hepatosomatic index; IW, initial weight; SGR, specific growth rate; WG, weight gain.

## Data Availability

The data that support the findings of this study are available from the corresponding author upon reasonable request.
